# Developments in Mannose‐Based Treatments for Uropathogenic *Escherichia coli*‐Induced Urinary Tract Infections

**DOI:** 10.1002/cbic.202000406

**Published:** 2020-11-02

**Authors:** Natasha E. Hatton, Christoph G. Baumann, Martin A. Fascione

**Affiliations:** ^1^ York Structural Biology Lab, Department of Chemistry University of York Heslington Road York YO10 5DD UK; ^2^ Department of Biology University of York York YO10 5DD UK

**Keywords:** anti-adhesion, carbohydrates, FimH, mannose, urinary tract infections (UTIs)

## Abstract

During their lifetime almost half of women will experience a symptomatic urinary tract infection (UTI) with a further half experiencing a relapse within six months. Currently UTIs are treated with antibiotics, but increasing antibiotic resistance rates highlight the need for new treatments. Uropathogenic *Escherichia coli* (UPEC) is responsible for the majority of symptomatic UTI cases and thus has become a key pathological target. Adhesion of type one pilus subunit FimH at the surface of UPEC strains to mannose‐saturated oligosaccharides located on the urothelium is critical to pathogenesis. Since the identification of FimH as a therapeutic target in the late 1980s, a substantial body of research has been generated focusing on the development of FimH‐targeting mannose‐based anti‐adhesion therapies. In this review we will discuss the design of different classes of these mannose‐based compounds and their utility and potential as UPEC therapeutics.

## Graphical abstract

**Keeping tracts on track**: The FimH adhesion protein has long been identified a potential therapeutic target for UPEC‐induced UTIs. As such, a substantial body of research has been conducted into the development of a mannose‐based FimH antagonist. Herein, we discuss the rationale behind this targeting approach and the three main categories of mannose‐based FimH inhibitors.

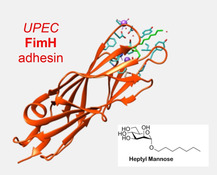

## 1 Introduction

### 1.1 Structure of the urinary tract, urothelium and uroplakins

The primary function of the urinary tract is to collect, transport, store and remove urine from the body, eliminating toxic waste products and metabolites generated by the kidneys.^[3]^ The urinary tract can be anatomically categorized into two subsections: the upper urinary tract (e. g., the kidneys and the ureters)^[4]^ and the lower urinary tract (e. g., the bladder and the urethra).^[4]^ The surface of the urinary tract is lined with a specialized epithelium known as urothelium.^[3]^ The biochemical and morphological features of the urothelium vary depending on its location within the urinary tract.^[3]^

**Figure 1:**
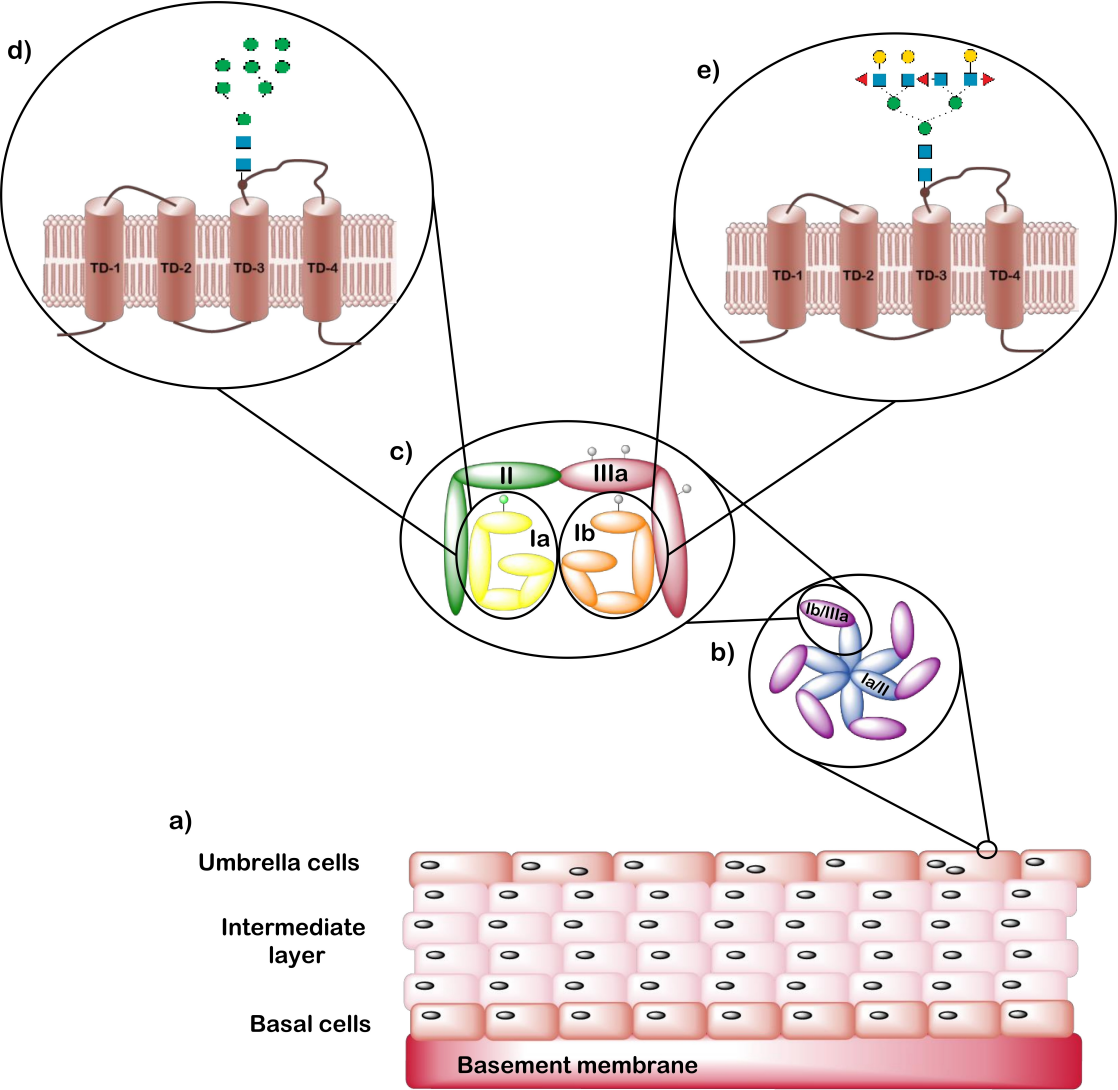
a) Structure of the urothelium with basal cells attached to a basement membrane, an intermediate layer and a layer of umbrella cells. b) Structure of the uroplakin (UP) plaque consisting of six heterodimeric units with each unit composed of two dimers UPIa/II and UPIb/IIIa. c) Structure of the heterodimer units in which a green ellipse represents a high‐mannose‐containing N‐glycan and the grey ellipses on UPIb/IIIa represent complex N‐glycans. d) Structure of UPIa and e) structure of UPIb. Both structures consist of four transmembrane domains and have an approximate molecular weight of 30 kDa.^[9]^ The green circles represent mannose residues, the blue squares represent glucosamine residues and the yellow circles represent galactose residues.

Generally, the urothelium is composed of three different layers; a basal cell layer attached to the basement membrane, an intermediate layer and an apical layer consisting of large hexagonal cells known as umbrella cells (Figure 1a).^[5]^ Umbrella cells are multinucleated, highly differentiated and accumulate a large amount of uroplakin proteins on their surface. This accumulation leads to the forming of a two‐dimensional plaque,[^3^, ^6^] which acts as a barrier to water and other toxic materials in the urine.^[7]^ In humans there are four different uroplakin (UP) proteins: UPIa, UPIb, UPII and UPIIIa.^[8]^ These units come together to form a heterodimer (Figure 1b) with six of these heterodimers combining to form the uroplakin plaque (Figure 1c).^[5]^ UPIa and UPIb belong to the tetraspanin family,^[5]^ consisting structurally of four rod‐like transmembrane domains. The first and second transmembrane domains are connected through a small extracellular loop, with a second large extracellular loop connecting the third and fourth transmembrane domains. The main difference between the two uroplakins is UPIa contains a high mannose glycan attached to the second extracellular domain at residue Asn169 (Figure 1d). In comparison, UPIb contains a tetraantennary fucosylated complex glycan attached to the second extracellular domain at the Asn131 residue (Figure 1e).^[5]^ UPII and UPIIIa are each structurally composed of a single transmembrane domain.

### 1.2 Urinary tract infections

Urinary tract infection (UTI) is the third most common type of infection experienced by humans after respiratory and gastro‐intestinal infections.^[10]^ Most UTI cases affect the lower urinary tract^[11]^ and can be either symptomatic or asymptomatic,^[12]^ with symptoms including increased urination frequency, pain during urination and blood in the urine.^[13]^ If left untreated UTIs can result in kidney damage, allowing bacteria to enter the blood stream resulting in urosepsis. Urosepsis accounts for 5–7 % of severe sepsis cases reported, with a mortality rate of between 25 and 60 %.^[14]^

Women are significantly more likely to experience a UTI than men^[15]^ due to the female urethrae being significantly shorter than a males (4 cm vs 20 cm).^[16]^ Approximately 40–50 % of women will experience a symptomatic UTI within their lifetime, with over half of these women suffering a relapse within six months.^[17]^ Age is another well‐recognized risk factor, with UTIs being the second most common form of infection in the non‐institutionalized elderly population, accounting for 25 % of infection cases.^[15]^ Due to their weakened immune systems approximately 10 % of males and 20 % of females over the age of 80 suffer from an asymptomatic UTI.^[18]^ Other risk factors for UTIs include urinary catheterization and diabetes.^[19]^

UTIs are caused by the invasion of foreign pathogens, with uropathogenic *Escherichia coli* (UPEC) being responsible for 80 % of cases. *Staphylococcus saprophytics* accounts for a further 10–15 %, and the remaining cases are caused by *Klebsiella, Enterobacter*, and *Proteus* species.^[20]^ UTIs can be classed as uncomplicated or complicated. For a UTI to be classed as complicated the patient must also suffer from either an underlying illness such as diabetes, a structural malformation of the urinary tract, or an obstruction of urine flow.^[21]^ Complicated UTIs are generally more difficult to treat,^[20]^ meaning the infections are often chronic with several different Gram‐positive and Gram‐negative bacteria present.

Currently UTIs are treated with a course of antibiotic such as Nitrofurantoin or Trimethoprim.^[18]^ However, an increasing problem observed in the treatment of UTIs is antibiotic resistance ‐ studies demonstrate UPEC strains contain over 30 different resistance genes to trimethoprim, with clinical resistance occurring in 16.7 % of cases.^[22]^ Nitrofurantoin is still active against *E. coli*, with fewer cases of resistance being reported than with Trimethoprim. However, Nitrofurantoin has a higher incidence of significant side effects like pulmonary fibrosis,^[23]^ and it is predicted that resistance to both antibiotics will increase. The enduring challenge of antibiotic resistance means that researching new effective treatments for UPEC‐induced UTIs is a clinical priority.^[22]^

### 1.3 Uropathogenic *E. coli* pathogenesis pathway

UPEC is responsible for the majority of reported uncomplicated UTI cases,^[17]^ thus identifying new targets within UPEC could serve as the basis for developing new treatments for both acute and recurrent UTIs.

**Figure 2:**
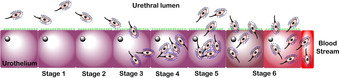
The pathogenesis cycle for UPEC consists of six stages: Stage 1) colonization of the periurethral areas and the urethra, Stage 2) movement of UPEC up the urethra, Stage 3) UPEC adherence, Stage 4) biofilm formation, Stage 5) epithelial cell invasion and formation of an intracellular bacterial population, and Stage 6) colonization of the urinary tract and kidneys by UPEC followed by entry into the blood stream.

The six stages of UPEC pathogenesis are summarized in Figure 2.^[24]^ The bacteria initially colonize the periurethral areas and the urethra, travelling up the urethra while growing as planktonic cells in the urine. While in the urinary tract, UPEC interact with and adhere to the urothelium. Once adhered, UPEC grows on the surface of the umbrella cells of the urothelium forming a biofilm, facilitating invasion of the epithelial cells. Once within the umbrella cells UPEC can begin multiplying, forming an intracellular bacterial population (IBC); this allows for further formation of a quiescent intracellular reservoir (QIR).^[25]^ UPEC can then invade the intermediate layers of the urothelium and lay dormant. These bacteria are protected from antibiotic treatment, making them extremely difficult to eliminate and thus the source of many recurrent infections.^[26]^ If untreated, UPEC will continue to colonize up the urinary tract, progressing to the kidneys.^[25]^ This colonization can result in kidney tissue damage and provides UPEC access to the blood stream, resulting in urosepsis.

**Figure 3:**
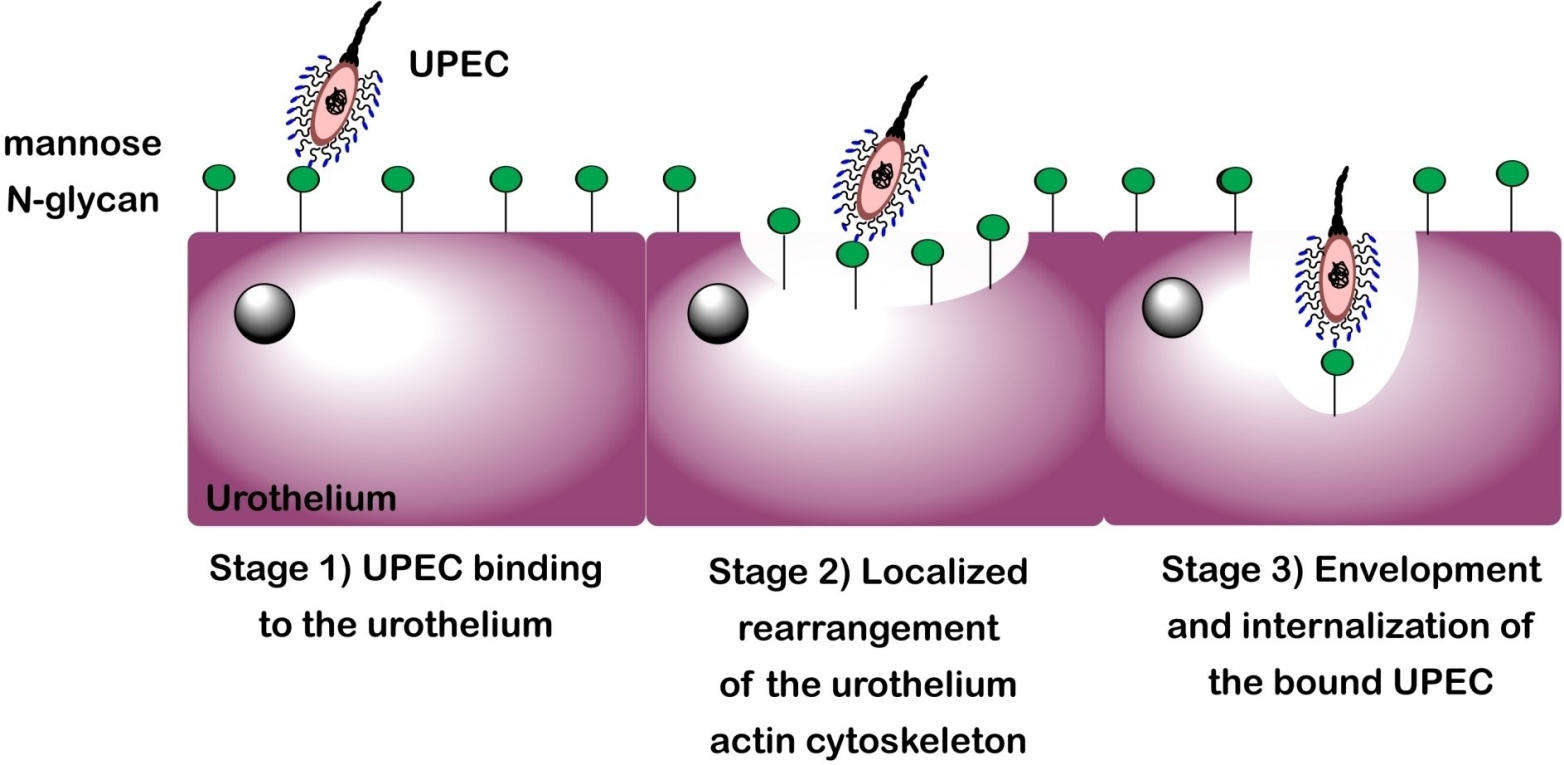
Schematic diagram showing the three‐stage membrane zippering mechanism thought to be used by UPEC during the invasion of the urothelium; Stage 1) binding of UPEC to the urothelium, Stage 2) localized rearrangement of the urothelium actin cytoskeleton, Stage 3) envelopment and internalization of the bound UPEC.

Invasion of the urothelium by UPEC occurs via a membrane zippering mechanism.^[27]^ This mechanism is stimulated by UPEC binding to the urothelium, which activates a complex signalling cascade, resulting in localized rearrangement of the urothelium actin cytoskeleton.^[27b]^ The cytoskeleton rearrangement leads to the envelopment and internalization of the bound UPEC (Figure 3). This complex signalling cascade has been shown to be reliant on many factors, such as focal adhesions; for example, Src,^[28]^ phosphoinositide 3‐kinase,^[27b]^ Rho‐family GTPases; actin bundling and adaptor proteins, for example, α‐actinin and vinculin;[^27b^, ^29^] lipid raft components, for example, caveolin‐1;^[30]^ and microtubules. Treatment of a host cell with a microtubule‐disrupting agent, such as nocodazole or vinblastine, has been shown to inhibit host cell invasion by UPEC.^[31]^

**Figure 4:**
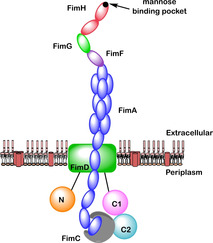
Structural organisation of the type 1 pilus which includes the following subunits: FimA (blue), FimC (grey), FimD (green rectangle), FimF (purple), FimG (green oval), and FimH (red). The position of the N‐terminal domain (N, orange) and two C‐terminal domains (C1, pink; C2, cyan) relative to the transmembrane pore in FimD are also indicated.

Adhesion of UPEC to the urothelium is mediated by UPEC binding to terminal d‐mannose units on UPIa. Without adhesion to the sugar UPEC would remain free in the urine and be removed from the bladder during urination, preventing the initial UPEC infection from progressing into a symptomatic UTI. To bind to terminal mannose units UPEC produce multiple 3‐μm‐long rod‐like structures on their surface known as type 1 pili (Figure 4).^[32]^ The type 1 pilus consists of multiple different subunits, including repeating units of the FimA protein, which form a 7 nm‐thick right‐handed helical rod. This rod is joined to a 3‐nm‐thick distal tip fibrillum, which itself is composed of three further subunits: two adapter proteins; FimF and FimG; and an adhesion protein, FimH.^[32]^

The exact process by which the type 1 pilus adheres to the urinary tract was unknown for a long time. One of the original theories proposed was that adhesion was mediated by the repeating FimA units which comprised most of the type one pilus.^[33]^ However, it is now recognized that the role of the repeating FimA units is to provide a structural scaffold.^[34]^ A wealth of research conducted in the late 1980s discovered that the minor subunits, FimF, FimG and FimH, were involved in adhesion.[^35^, ^36^] Christiansen and co‐workers found that recombinant bacteria lacking either all three subunits or just FimH displayed no ability to bind to erythrocytes, indicating that FimH plays a critical role in adhesion.^[37]^ Klemm and co‐workers^[38]^ confirmed this observation, providing direct evidence that FimH is responsible for mannose‐mediated adhesion.^[38]^

### 1.4 FimH structure and catch bond mechanism

**Figure 5:**
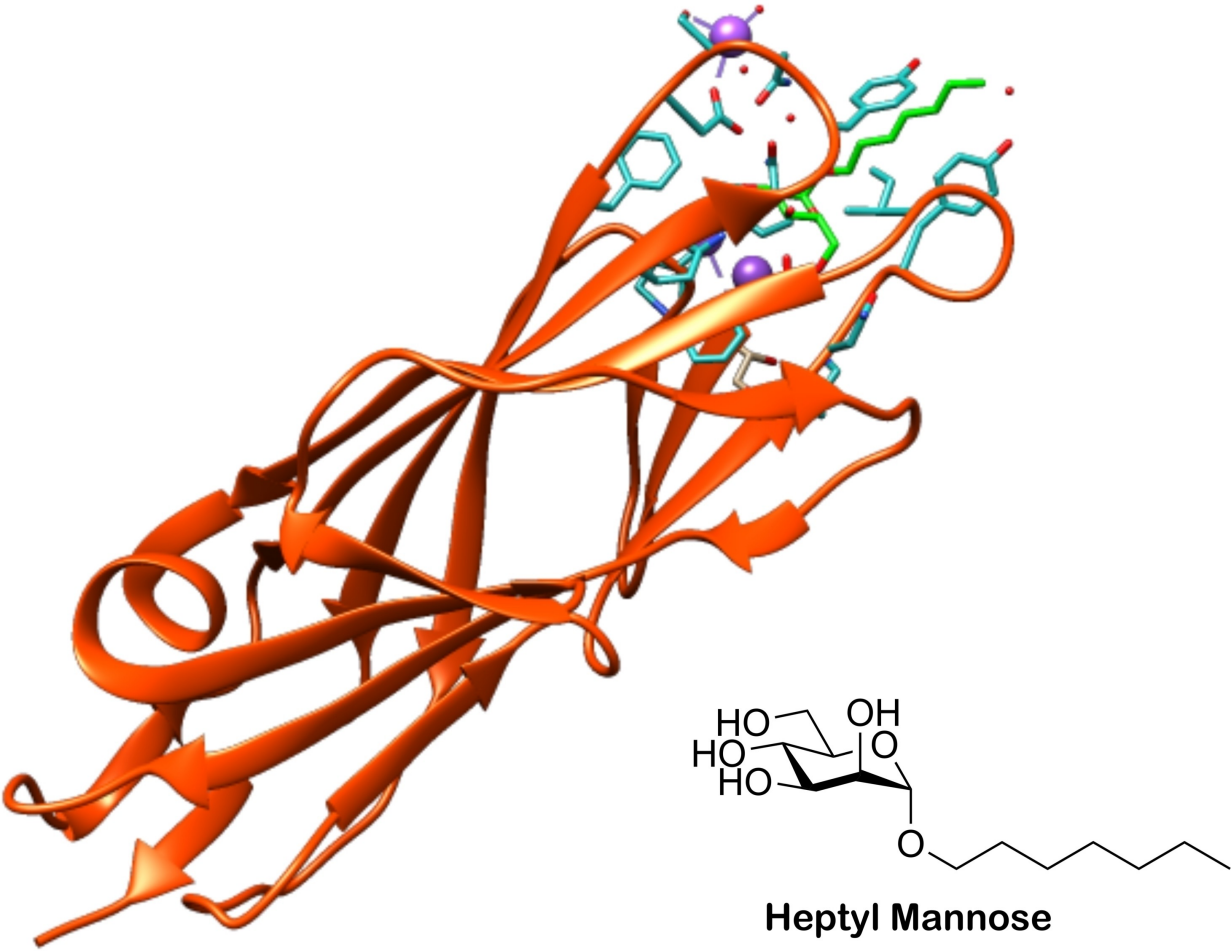
Crystal structure of FimH (orange) with a heptylmannoside (green) ligand bound in the N‐terminal lectin domain (FimH_LD_; PDB ID: 4LOV).^[2]^ Residues with side chains shown as sticks (blue) are involved in noncovalent interactions with heptylmannoside, sodium ions (purple spheres) or water molecules (red spheres). The dashed lines (purple) denote short‐range, noncovalent interactions.

FimH is comprised of two domains (Figure 5). The first is a C‐terminal pilin domain (FimH_PD_), which attaches FimH to the pilus rod^[39]^ through the neighbouring subunit FimG. This attachment occurs by donor strand complementation,^[40]^ and is an effective way of providing strong intermolecular linkages through the donation of one β‐strand from one subunit (e. g., FimG) into the β‐sandwich of the neighbouring subunit (e. g., FimH_P_).^[41]^ The second FimH domain is the N‐terminal lectin domain (FimH_LD_), which contains a mannose‐binding pocket^[39]^ that can bind to mannose sugars on the urothelium, mediating adhesion of UPEC to the urinary tract.^[32]^

**Figure 6:**
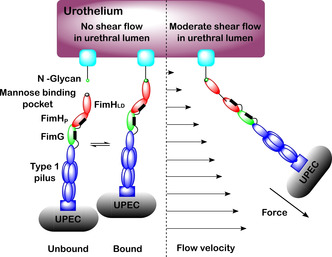
Catch bond mechanism for FimH binding to mannose in urethral lumen under no shear flow conditions, where unbound and bound forms exist at equilibrium, and moderate shear flow conditions, where the bound state is favoured. Under moderate shear flow conditions, the hydrodynamic drag on the micron‐sized UPEC bacterium (not to scale) results in a physical force on the tether which activates the catch bond mechanism in FimH. The higher affinity binding to mannose under these conditions prevents the UPEC bacterium from being flushed out of the urinary tract during urination.

The two‐domain structure of FimH allows the type‐1 pilus to form a catch‐bond when bound.^[40]^ A catch‐bond is a type of noncovalent interaction in which the dissociation lifetime of the bond increases when force is applied^[42]^ (Figure 6). Wirtz and co‐workers provided the first unequivocal evidence for the existence of catch‐bonds.^[43]^ Prior to this, catch bonds were treated as one potential explanation for why the adhesion affinity of some bacteria (e. g., UPEC) increases in moderate shear flow. An alternative theory was the transport limiting binding model. This model, which has fallen out of favour, suggests that increased shear flow causes an increase in both dissociation and association rates, thus an enhancement in bacterial adhesion would have to be caused by an increase in bacterial association not a decrease in bacterial dissociation.^[44]^

The catch‐bond in FimH is biphasic, meaning under moderate force (such as that experienced by the sides of the urinary tract during urination) FimH binds to ligands with higher affinity, whereas exposure to higher shear flow leads to a decrease in binding affinity due to an increase in the dissociation rate for the FimH‐ligand interaction.^[42]^ Moderate mechanical force induces the separation of the FimH_LD_ and FimH_P_ subunits (Figure 6). This separation switches the lectin domain from a low affinity state to a high affinity state^[45]^ reducing the rate of spontaneous ligand release, resulting in a 1000‐fold higher affinity of FimH for mannose sugars under moderate flow conditions compared with static conditions.^[40]^ The relatively weaker affinity of FimH under static conditions favours invasion of UPEC along the urinary tract during static conditions, while the high affinity of FimH under moderate flow conditions enables UPEC to be retained in the urinary tract during urination. Glockshuber and co‐workers^[40]^ investigated how the different flow conformation states affect the binding of FimH to *N*‐glycans. A mixture of synthetic α‐linked mono‐ and dimannosides were used to represent the natural terminal α‐d‐mannoside moieties present on FimH targeted glycoproteins in the bladder.^[40]^ Using these ligands, kinetic and structural characterization of the binding properties of FimH under both static and flow conditions could be investigated. It was demonstrated that the increased affinity of FimH under flow conditions compared to static conditions was ligand independent.^[40]^ Glockshuber and co‐workers further found that dimannosides bound with higher affinity compared to monosaccharides, with the difference in affinity determined by the rate of spontaneous ligand dissociation.^[40]^ Under static conditions FimH binds to all natural terminal α‐d‐mannoside structures with medium affinity, while under flow conditions FimH binds to all d‐mannosides at a 2000‐fold higher affinity with a 70,000‐fold decrease in ligand dissociation rate and 30‐fold increase in ligand association rate.^[40]^ Glockshuber and co‐workers additionally found that even though FimH favors monovalent *N*‐glycan binding when *N*‐glycans are in short supply, each *N*‐glycan can bind up to three FimH units at once.^[40]^

### 1.5 Extended FimH binding pocket

**Figure 7:**
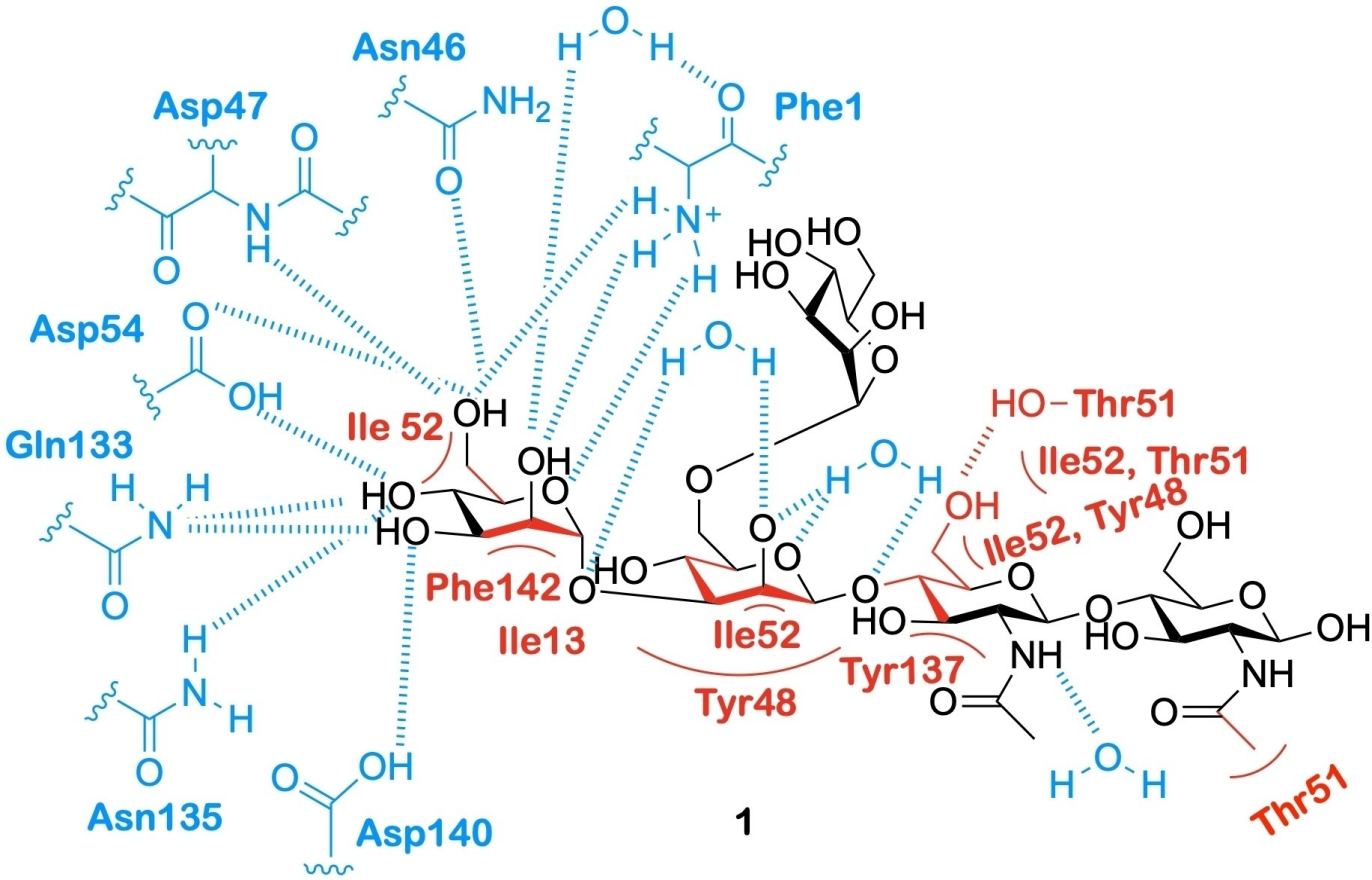
A depiction of the main interactions that occur between mannose‐based pentasaccharide **1** and the extended FimH binding site.^[46]^ Red indicates interactions mediated by van der Waals, aromatic stacking and hydrophobic interactions, blue indicates interactions mediated by hydrogen bonding.

The FimH lectin domain contains 157 amino acids, which assemble to form an 11‐stranded β‐barrel structure.^[39]^ Encompassed within the β‐barrel structure is a polar binding pocket^[40]^ (residues Asn46, Asp47, Asp54, Gln133, Asn135 and Asp140) to which terminal mannose units can engage in a complex network of hydrogen bonding and electrostatic interactions.^[39]^ The polar binding pocket is surrounded by a hydrophobic region, (residues Phe1, Ile13 and Phe142)^[39]^ which contains a tyrosine gate (residues Tyr48, Ile52, Thr51 and Tyr137).^[39]^ This region provides support for the binding site through electrostatic interactions with the tyrosine gate, which is shown to be influential in the ability of ligands to enter the binding site.^[46]^ Further interactive features of the FimH lectin domain are a small hydrophobic pocket^[47]^ adjacent to the sugar binding pocket (residues Ile52, Tyr137 and Asn138),^[39]^ a salt bridge (residues Arg98 and Glu50) which facilitates further hydrogen bonding,^[47]^ and the Tyr48 and Tyr137 residues, which partake in hydrophobic and ring stacking interactions[^47^, ^48^] as well as forming direct and water‐mediated hydrogen bonds to ligands.^[48]^ A summary of the main interactions that occur between the FimH lectin domain and mannose‐based pentasaccharide **1** is presented in Figure 7.

**Figure 8:**
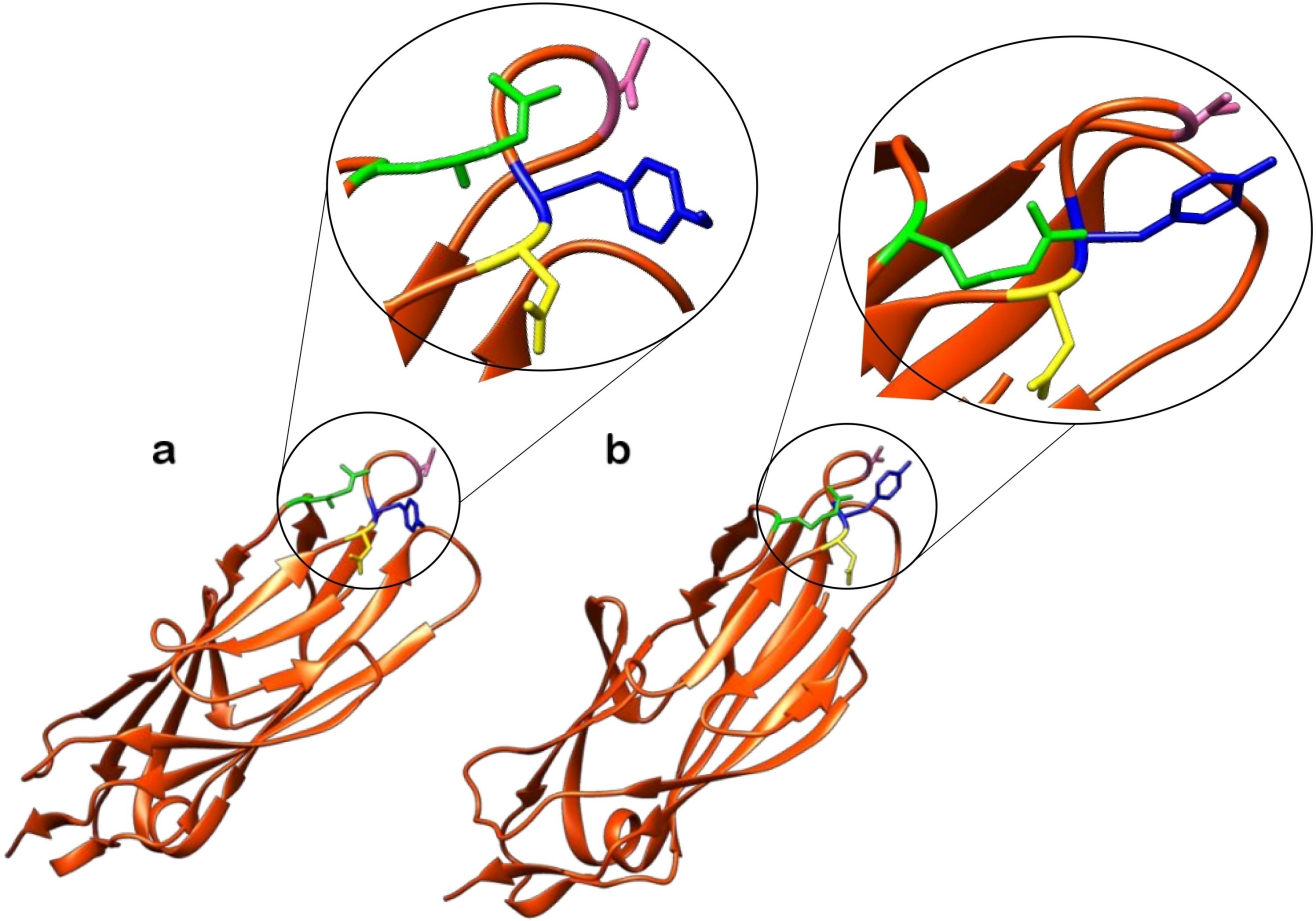
a) Structure of FimH the lectin domain with the tyrosine gate open (PDB ID: 4AV0).^[51]^ b) Structure of the FimH lectin domain when the tyrosine gate is closed (PDB ID: 4ATT).^[52]^ Note the movement of the Tyr48 residue (blue) from facing the Asp47 residue (yellow) in the open conformer to facing the Thr51 residue (pink) in the closed conformer. The Arg98 residue (green) plays a role in stabilizing the surface loop conformation on which the Thr51 residue is located.

As depicted in Figure 7, the binding of a mannose‐based ligand to the extended FimH binding site is the result of a complex network of interactions and hydrogen bonding. As such, small changes to the structure of the ligand can disrupt this binding network, resulting in either a decrease or complete loss of ligand affinity. Epimers of mannose (e. g., glucose and galactose) exhibit no inhibitory effects on FimH,[^39^, ^49^] due to the lack of an axial C‐2 hydroxy group preventing them from partaking in critical interactions with the polar binding pocket. The five‐membered sugar fructose, which like mannose contains an axial C‐2 hydroxy group, has been shown to have weak activity for FimH.[^39^, ^49a^, ^49b^] A further stereochemical requirement exhibited by the FimH lectin domain is that the terminal mannose unit must be in the alpha orientation.^[50]^ Terminal α‐mannosides can engage in water‐mediated hydrogen bonding within the FimH lectin domain;^[39]^ these interactions would not be possible if the mannoside was in the β‐configuration. A further characteristic of the extended FimH binding site that facilitates critical interactions with ligands are its hydrophobic residues. These residues surround the mannose binding pocket and consists of the hydrophobic support and tyrosine gate residues as well as residue Thr51, which extends out of the hydrophobic ridge along a hydrophobic groove.^[39]^ Unlike the polar binding pocket, the tyrosine gate structure is flexible, interacting with ligands by a mixture of π‐stacking and van der Waals interactions.^[39]^ The flexibility of the tyrosine gate is the result of the Tyr48 residue being able to rotate, allowing for three different tyrosine gate conformations; open, closed and half open.^[51]^ In its unbound resting state FimH adopts an open conformation; here the side chain of the Tyr48 residue is positioned inwards facing residues Asp47 and Arg98 (Figure 8a).^[51]^ In the closed gate conformation the Tyr48 residue shifts alignment towards the Thr51 residue (Figure 8b).^[52]^ If the side chain of the Tyr48 residue is aligned somewhere between the open and closed position, then the gate is classified as half open.^[51]^ When bound to a ligand the tyrosine gate can either remain in its open conformation or shift to the half open or closed conformation.^[51]^ A conformational change often accompanies ligand binding in order to minimize Gibbs free energy, thus different ligands will bind to different conformational states.[^51^, ^52^] When a ligand is flexible and can extend out of the binding site like some oligomannosides,^[46]^ the open conformation is adopted as this allows the second mannose residue in the oligosaccharide to interact with the Tyr48 residue of the binding site.^[47]^ When a ligand contains a sterically rigid aglycone group (e. g., 1,4‐biaryl group) the tyrosine gate will shift to the closed conformation, positioning the aglycone residue outside of the tyrosine gate. This conformational shift is favored by the formation of strong π‐stacking interactions between the aglycon and the outer side of the aromatic ring on the Tyr48 residue.[^47^, ^53^]

## 2 Mannose‐Based FimH Inhibitors

This review will survey and critically assess recent progress in the development of FimH targeting mannose‐based analogues with potential applications against UPEC‐induced UTIs. The review will focus on three categories of mannose‐based FimH inhibitors: oligosaccharide‐based FimH inhibitors (Section 2.1), α‐d‐mannopyranoside‐based inhibitors (Section 2.2.) and polyvalent mannose‐based inhibitors (Section 2.3). Note that informative reviews on this subject have previously been published.[^39^, ^54^]

### 2.1 Oligosaccharide‐based FimH inhibitors

FimH primarily targets large branched mannose‐capped oligosaccharides found on N‐glycosylated proteins which line the urinary tract^[39]^ such as UPIa.^[55]^ The preference of FimH for mannose‐capped oligosaccharides can be seen in ligand affinity assays, with α‐d‐mannose‐capped oligosaccharides displaying 100–200 times greater affinity for FimH than α‐d‐mannose monomers.^[39]^ This increase in affinity is due to mannose‐based oligosaccharides being able to interact with the extended FimH binding site.

**Figure 9:**
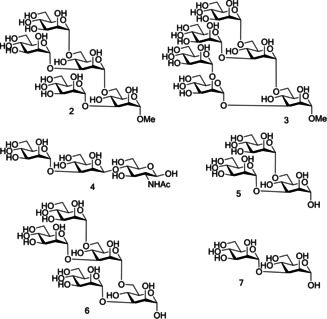
Comparison of the structures of potent oligosaccharide‐based FimH inhibitors (oligosaccharides **2**, **3**, and trisaccharide **4**) to weak oligosaccharide‐based FimH inhibitors (mannotriose **5**, mannopentaose **6** and Manα‐1,3Man **7**).

Due to their role as natural FimH ligands and their ability to act as FimH inhibitors, much research has been performed into using mannose‐capped oligosaccharides as a curative treatment for UPEC‐induced UTIs. Sharon and co‐workers^[56]^ investigated the ability of a large range of mannose glycosides and mannose‐capped oligosaccharides to inhibit *E. coli*‐induced yeast aggregation.^[56]^ The three most potent inhibitors were oligosaccharides **2**, **3** and trisaccharide **4** (Figure 9),^[56]^ with research suggesting that trisaccharide **4** is the optimal size for binding to the extended FimH binding site.^[56]^ Further investigation found that the high binding affinity displayed by trisaccharide **4** was due to a few key structural components. Firstly, trisaccharide **4** contains a Man‐α1,3‐Man linkage at the non‐reducing terminus; this linkage has been shown to be highly preferential in FimH binding.^[57]^ A second beneficial structural feature is the presence of a *N*‐acetyl glucosamine sugar, as neither mannotriose **5** nor mannopentaose **6** (Figure 9) exhibited significantly higher FimH binding affinity than Man‐α1,3‐Man **7** (Figure 9).^[57]^ Furthermore, docking studies suggest that the structure of trisaccharide **4** allows both the central mannose unit and the GlcNAc unit to interact with the tyrosine gate, accounting for the high affinity displayed by trisaccharide **4**.^[57]^

**Figure 10:**
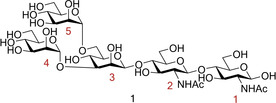
Structure of the potent FimH inhibitor oligomannoside **1**.

A further oligosaccharide with high affinity to FimH is oligosaccharide **1** (Figure 10, *K*_d_=20 nM)^[57]^), displaying a tenfold increase in affinity compared to Man‐α1,3‐Man.[^46^, ^57^] Oligosaccharide **1** contains a terminal chitobiose unit; these units are ubiquitous in nature, providing a bridge between mannosides and asparagine residues in the Asn‐X‐Ser/Thr motif found in N‐linked glycoproteins.^[46]^ Chitobiose units have been shown to interact with the extended FimH binding site. A crystal structure of oligomannoside **1** bound to the extended FimH receptor binding domain identified some of the key binding interactions.^[46]^ Firstly, Man4 at the nonreducing end was shown to be anchored into the polar binding pocket. Further interactions were mediated through the tyrosine gate interacting with the Man‐α1,3‐Man‐β1,4‐GlcNAc backbone via the α1,3 and the first β1,4 glycosidic linkages. The second mannose unit at the non‐reducing end, Man5, was not directly recognized by FimH due to the unit partially extending out of the binding site. GlcNAc1 was also shown to partially exit the binding site, folding over residue Thr51. Man5 and GlcNAc1 displayed the most flexibility of all the saccharide units,^[46]^ likely due to being the least closely bound to the extended FimH binding site. This crystal structure provides further evidence that trisaccharide **4** may construct the optimal motif for FimH binding, containing the critical Man‐α1,3‐Man‐β1,4‐GlcNAc backbone needed for interactions with the extended FimH binding site.

The oligomannoside structures discussed here were obtained by a number of different methods including chemical synthesis, isolation from yeast extract and isolation from the urine of patients suffering from mannosidosis and GM1 gangliosidosis. ^66[46,57]^

Chemical synthesis is one of the most common methods used to obtain mannose‐capped oligosaccharides. As is common in carbohydrate synthesis, the synthesis of these oligosaccharides relies on a complex scheme of protection, glycosylation and deprotection steps. To further add to this complexity, potent FimH inhibitors such as trisaccharide **4** and oligosaccharide **1** contain a critical β‐mannosidic bond between the central mannose unit and the GlcNAc unit. β‐Mannosidic bonds have long been recognised as the most difficult linkages to construct for carbohydrate chemists.^[58]^ Over the last 25 years multiple groups have conducted a wealth of research into the development of β‐mannosidic bonds, developing synthetic methods to afford β‐mannosides in a high yielding and selective manner.[^58^, ^59^] Despite this research, the requirement for pre‐activation and need for specific mannoside donor protection groups (e. g., *O*‐4,6 benzylidene protection)^[60]^ still makes β‐mannoside synthesis challenging, and consequently many opt to prepare simpler α‐mannoside counterparts. A further factor restricting the use of mannose capped oligosaccharides as therapeutics is their size which makes them unlikely to be orally absorbed. Lack of oral absorption coupled with synthetic complexity, has therefore restricted the application of mannose‐capped oligosaccharides, and as such many research groups have chosen to focus on the more easily synthesized α‐d‐mannopyranoside‐based inhibitors as FimH antagonists.

### 2.2 α‐d‐Mannopyranoside‐based inhibitors

**Figure 11:**
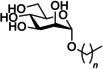
Structure of the alkyl mannoside scaffold where *n*=0–7.

One of the first classes of d‐mannopyranoside‐based FimH inhibitors were alkyl mannosides (Figure 11, Table 1 entry 1). These were discovered serendipitously when De Greve and co‐workers found butyl‐α‐d‐mannoside occupied the mannose binding site.^[61]^ Investigations found butyl‐α‐d‐mannoside (*K*_d_=151 nM) to bind to FimH with 15–30 times greater affinity than α‐d‐mannose (*K*_d_=2.3 μM).^[61]^ Further investigation into the binding affinity of alkyl mannosides found that in general binding affinity increases as alkyl chain length increases, with the binding affinity peaking for hept‐d‐mannoside (*K*_d_=5 nM).^[61]^ One theory explaining this trend is that increasing the chain length increases the van der Waals interactions with the hydrophobic groove and tyrosine gate, but once the alkyl chain is longer than a heptyl chain it extends beyond the hydrophobic region into solvent‐exposed areas,^[39]^ increasing the free energy of binding and thus lowering binding affinity.

**Table 1:** A comparison of the α‐d‐mannopyranoside‐based inhibitors discussed.

		Structure	Refs.
1)	alkyl mannosides	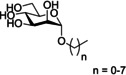	^[61]^
2)	aryl mannosides	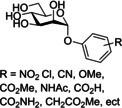	[^47^, ^63^, ^64^, ^65^]
3)	biphenyl mannosides	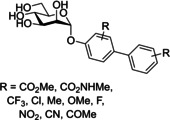	[^1b^, ^47^, ^53^, ^66^]
4)	squarate mannosides	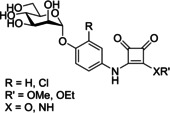	[^64^, ^68^]
5)	septanoses	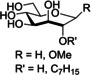	^[69]^
6)	thiazolylaminomannosides (TazMan) and neothiazolylaminomannosides (NeoTazMan)	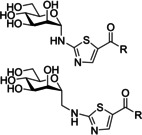	^[70]^
7)	indolynyl‐mannosides	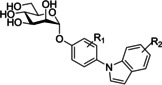	^[71]^

**Scheme 1:**
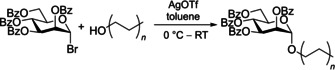
Reaction scheme for the synthesis of alkyl mannosides by glycosylation with aliphatic alcohols using an AgOTf activation system.

The alkyl mannosides used by De Greve and co‐workers^[61]^ were synthesized using a procedure reported by Tidén and co‐workers^[62]^ for the synthesis of octyl and tetradecyl mannosides. Here alkyl alcohols underwent silver triflate‐promoted glycosylations to 2,3,4,6‐tetra‐*O*‐benzoyl‐α‐d‐mannopyranosyl bromide (Scheme 1), followed by Zemplén deacylation to afford the deprotected alkyl mannoside ligand.

**Figure 12:**
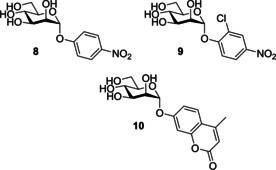
Structure of p‐nitrophenyl‐α‐mannoside (8), p‐nitro‐O‐chlorophenyl‐α‐mannoside (pNoClPαMan; 9) and 4‐methylumbelliferyl‐α‐mannoside (MeUmbαMan; 10).

Sharon and co‐workers^[63]^ reported aromatic α‐mannosides (Table 1, entry 2) to be powerful inhibitors of the adherence of type 1 fimbriated *E. coli* to both yeast and intestinal epithelial cells. With *p*‐nitrophenyl‐α‐mannoside **8** (Figure 12) shown to be approximately 70 times more effective than methyl α‐mannoside for both inhibiting yeast agglutination by *E. coli* 025 and inhibiting the adherence of *E. coli* 0128 to guinea pig ileal epithelial cells.[^56a^, ^63^] The two most potent aromatic α‐mannoside inhibitors reported by Sharon and co‐workers[^56a^, ^63^] were *p*‐nitro‐*O*‐chlorophenyl‐α‐mannoside (*p*N*o*ClPαMan) **9** and 4‐methylumbelliferyl‐α‐mannoside (MeUmbαMan) **10** (Figure 12). These analogues were shown to increase the inhibition of yeast agglutination by *E. coli* 025 by a factor of 717 (*p*N*o*ClPαMan **9**) and 600 (MeUmbαMan **10**) compared to methyl α‐mannoside. A significant increase in inhibition of *E. coli* 0128 adherence to guinea pig ileal epithelial cells was also observed [470 times for (*p*N*o*ClPαMan **9**) and 1015 times for (MeUmbαMan **10**) with respect to methyl α‐mannoside]. This increase in affinity displayed by FimH for aromatic α‐mannosides compared with methyl α‐mannoside is likely due to the aromatic side chain being able to establish favourable π–π stacking interactions with the tyrosine gate.^[64]^

Building upon the high potency displayed by *p*‐nitrophenyl‐α‐mannoside, Lindhorst and co‐workers synthesized a series of *para*‐substituted aryl α‐d‐mannosides, with one analogue achieving significantly higher potency than *p*‐nitrophenyl‐α‐mannoside [relative inhibitory potential (RIP) of 1.6 vs 1].^[65]^ Further work into the development of an aromatic α‐mannoside‐based inhibitor was performed by Han and co‐workers.^[47]^ Here they synthesized a series of aryl‐substituted α‐d‐phenylmannosides, evaluating their biological activity using a guinea pig red blood cell‐based hemagglutination (HA) assay. This assay measures the concentration of inhibitor needed for >90 % HA inhibition, and this concentration is known as the hemagglutination inhibition (HAI) titre. The HAI titre of new compounds can be compared to that of other compounds in order to assess the potency of new compounds.

Han and co‐workers synthesized an extensive series of aryl substituted α‐d‐phenylmannosides functionalized with a variety of groups (Cl, NO_2_, CN, OMe, CO_2_Me, NHAc, CO_2_H, CONH_2_, CH_2_CO_3_Me) at either the *ortho*, *meta* or *para* position.^[47]^ Generally, it was noted that phenylmannosides functionalized at the *ortho* and *meta* positions gave better potency than phenylmannosides functionalized at the *para* position. The most potent phenylmannoside contained a methyl ester group at both *meta* positions, being three times more potent than the phenylmannoside containing a single *meta* methyl ester.^[47]^

**Figure 13:**
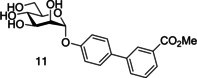
Structure of biphenyl mannoside 11.

Another well‐investigated group of FimH‐targeting analogues are biphenyl mannosides (Table 1, entry 3).[^47^, ^53^, ^66^] Han and co‐workers hypothesized that the potency of aryl mannosides could be increased by the addition of a second aryl ring. This hypothesis was explored using analogues containing an additional aryl ring system in conjugation with the parent ring of the aryl mannoside. The most potent analogue was biphenyl mannoside **11** (HAI=1 μM, IC_50_=0.94 μM)^[47]^ which contained a methyl ester at the *meta* position of the second ring system (Figure 13). A high resolution X‐ray crystal structure of biphenyl mannoside **11**, showed that both aromatic rings were able to partake in hydrophobic and π–π stacking interactions with the closed Tyr48 residue, with the methyl ester further able to hydrogen bond with the salt bridge (residues Arg98 and Glu50).^[47]^

**Figure 14:**
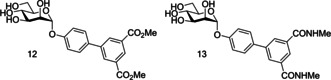
Structure of biphenyl mannoside 12 and biphenyl mannoside 13.

Further SAR evaluation of the terminal biphenyl mannoside aryl ring identified two potent biphenyl mannosides: biphenyl mannoside **12 (**HAI=0.15 μM) and biphenyl mannoside **13** (HAI=0.37 μM, IC_50_=0.74 μM;^[66a]^ Figure 14).^[47]^ Biphenyl mannoside **12** displayed good potency in the HAI assay, yet due to its insolubility could not be used in a biofilm assay.^[66b]^ However, biphenyl mannoside **13** was used in a biofilm assay and showed impressive activity, with an IC_50_ of 0.74 μM.^[66b]^ Initial dosing in mice established biphenyl mannoside **13** to be orally bioavailable and stable to metabolism, and the only metabolic degradation pathway detected was hydrolysis of the glycosidic bond. Metabolic degradation of biphenyl mannoside **13** should be minimal as >95 % was excreted in the urine unchanged. Moreover, no apparent toxicity was observed up to a dose of 200 mg/kg, using physiological changes and survival as assessment parameters. Further investigation using an adapted preclinical murine model suggested that biphenyl mannoside **13** was effective at preventing infections, treating established UTIs and increasing the activity of antibiotic treatments.^[66b]^

**Figure 15:**
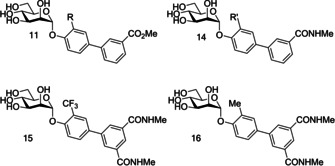
Structure of biphenyl mannoside **11**, biphenyl mannoside **14**, biphenyl mannoside **15** and biphenyl mannoside **16** where R=CF_3_, Cl, Me, OMe, F and R’=CF_3_, Me, Cl.

Han and co‐workers previous work on α‐d‐phenylmannosides showed that substitution at the *ortho* position with chloro and cyano groups could significantly increase potency.^[47]^ Using widely available biphenyl mannoside **11** as a scaffold (Figure 15), Han and co‐workers also synthesized a number of biphenyl mannosides substituted at the *ortho* position of the top ring, and observed the general potency trend CF_3_>Cl=Me>OMe>F.^[66a]^ One explanation for the increase in potency upon *ortho* substitution is that the presence of *ortho* substituents increases the hydrophobic contact with the tyrosine gate at residues Ile52 or Ile13. This explanation is supported by the potency trend seen above, as addition of a CF_3_ group would result in the largest increase in hydrophobic contact and thus the largest increase in potency. Further investigation was performed using biphenyl mannoside **14** (IC_50_=1.35 μM)^[66a]^ as a scaffold, with a similar potency trend observed: CF_3_>Me>Cl.^[66a]^ Two final analogues were synthesized; biphenyl mannoside **15** (HAI=0.01 μM, IC_50_=0.043 μM)^[66a]^ and biphenyl mannoside **16** (HAI=0.02 μM, IC_50_=0.073 μM)„^[66a]^ with both displaying increased potency to biphenyl mannoside **13** (HAI=0.37 μM).^[66a]^

**Figure 16:**
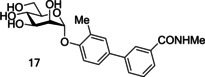
Structure of biphenyl mannoside **17**.

Biphenyl mannosides **15** and **16** displayed good potency, but their low log *p* values of −6.27 and −8.46, respectively, suggest they would have poor oral absorption.^[66a]^ Biphenyl mannoside **17** (Figure 16) had a significantly higher log *p* of −3.89, and therefore should have better oral absorption.^[66a]^ Pharmacokinetic studies in mice showed biphenyl mannoside **17** (IC_50_=0.16 μM)^[66a]^ to be a promising lead candidate, maintaining a concentration in the urine and plasma well above the predicted minimum effective concentration for over 6 h. Further *in vivo* tests used a chronic infection mouse model; this model uses C3H/HeN mice with chronic cystitis at two‐weeks post‐infection. The efficacy of compounds can be monitored by the number of colony forming units (CFU) present at selective time periods after compound administration.^[66b]^ Mice treated with biphenyl mannoside **17** showed a significant reduction of chronic cystitis six hours post‐treatment; however, by 24 hours post‐treatment the number of CFU had started to increase, but remained lower than in control mice.^[66b]^ This CFU increase was shown to be prevented by administering three doses of biphenyl mannoside **17** every eight hours. These *in vivo* experiments suggest that biphenyl mannoside **17** provided a promising lead compound which could be used in preclinical trials.^[66b]^

**Figure 17:**
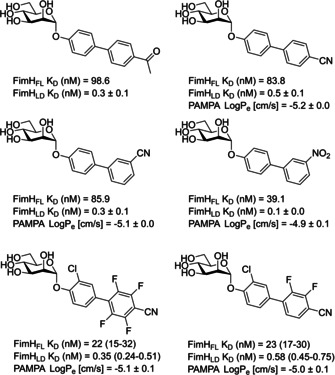
Structure of some substituted biphenyl mannoside analogues with promising FimH activity and pharmacokinetic properties,^[1]^ such as dissociation constants (*K*_D_) and log *p*_e_ values assessed using a parallel artificial membrane permeability (PAMP) assay.

A limitation of the antagonistic studies performed on previously designed biphenylmannosides and other α‐d‐mannopyranoside‐based inhibitors are the methods used; for example, a fluorescence polarization assay, an isothermal titration calorimetry (ITC)‐based assay, and a surface plasmon resonance (SPR)‐based assay. These methods rely on the use of FimH_LD_,^[1a]^ which is locked in the high‐affinity FimH conformation.^[2]^ As discussed previously, the FimH lectin can adopt three conformational states; an unbound low‐affinity conformation, a bound medium‐affinity conformation which occurs under static conditions, and a bound high‐affinity conformation which occurs under shear force.^[1a]^ Therefore, only measuring an antagonist affinity against one FimH conformation could limit the accuracy of the results. Antagonistic studies performed using most target‐based assays and cell‐based assays (e. g., hemagglutinin assays or a flow cytometry‐based assay) are not affected by these inaccuracies, as they use *E. coli* cells producing FimH_FL_, which can adopt all three conformers.^[1a]^ Monomeric FimH_FL_ is inherently unstable[^1a^, ^67^] and thus a native model must be used instead. This model is composed of FimH expressed as a biomolecular complex, where the incomplete fold of FimH_PD_ is complemented by a synthetic donor‐strand peptide. Subsequently, comparison of the binding affinities of a series of biphenyl analogues showed an approximate 100‐fold decrease in affinity from FimH_LD_ to FimH_FL_. Recent investigations into biphenyl mannoside affinity for FimH_LD_ and FimH_FL_ has led to the design of some promising substituted biphenyl mannoside analogues (Figure 17).^[1]^ These analogues display nanomolar potency against FimH_FL_ and sub‐nanomolar potencies against FimH_LD_, alongside promising pharmacokinetic properties.^[1]^

**Scheme 2:**
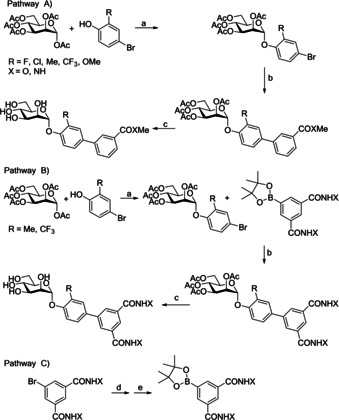
Synthesis of *ortho*‐substituted biphenyl mannosides. Pathways A and B: a) BF_3_ ⋅ Et_2_O, CH_2_Cl_2_, reflux, 45 h, (25–75 %); b) 3‐substituted phenylboronic acid derivatives, cat. Pd(PPh_3_)_4_, Cs_2_CO_3_, dioxane/water (5 : 1), 80 °C, 1 h; c) cat. MeONa, MeOH, RT, 12 h, (b+c 3–64 %): Pathway C: d) MeNH_2_/EtOH, RT, e) bis(pinacolato)diboron, cat. Pd(dppf)Cl_2_, KOAc, DMSO, 80 °C.

The *ortho*‐substituted biphenyl mannosides were synthesized via two routes (Scheme 2). Most analogues were synthesized using Pathway A. The first step of this pathway is Lewis acid mediated glycosylation of mannose pentaacetate with a 2‐substituted 4‐bromophenol analogue (step a). This is followed by a Suzuki cross‐coupling with a commercially available 3‐substituted phenylboronic acid derivative to give a protected *ortho*‐substituted 4′‐biphenyl mannoside (step b). The final step is a Zemplén deacetylation, affording the deprotected *ortho*‐substituted 4’‐biphenyl mannoside (step c). Synthesis of the diamide analogues was performed using Pathway B, following the same initial and final steps as Pathway **A** (step a and d), but using a 3,5‐di‐(N‐methylaminocarbonyl)‐phenylboronic acid pinacol ester synthesized in‐house (Pathway C) in the Suzuki cross‐coupling reaction (step d).^[66a]^

**Figure 18:**
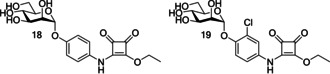
Structure of squarate mannosides **18** and **19**.

A further class of d‐mannopyranoside‐based inhibitors are squarate mannosides (Table 1 entry 4). Lindhorst and co‐workers used computer‐based docking methods to predict the binding affinities of a number of FimH inhibitors; for example, methyl mannose, *p*‐nitro‐phenyl‐α‐mannoside and two squarate mannosides (squarate mannoside **18** and squarate mannoside **19**, Figure 18). An enzyme‐linked immunosorbent assay (ELISA) was performed to test the inhibitors ability to inhibit type 1 fimbriae‐mediated bacterial adhesion, with methyl mannose used as a standard (RIP=1). Squarate mannosides **18** and **19** were shown to be the most potent inhibitors in the ELISA, with RIPs of 1600 and 6900, respectively.^[64]^ For the inhibitors analysed the expected inverse trend between docking score and RIP was observed. One notable exception was squarate mannoside **18**, which had a predicted FimH affinity higher than that of squarate mannoside **19**. However, the ELISA showed squarate mannoside **19** to be four times more potent that squarate mannoside **18**.^[64]^

**Scheme 3:**
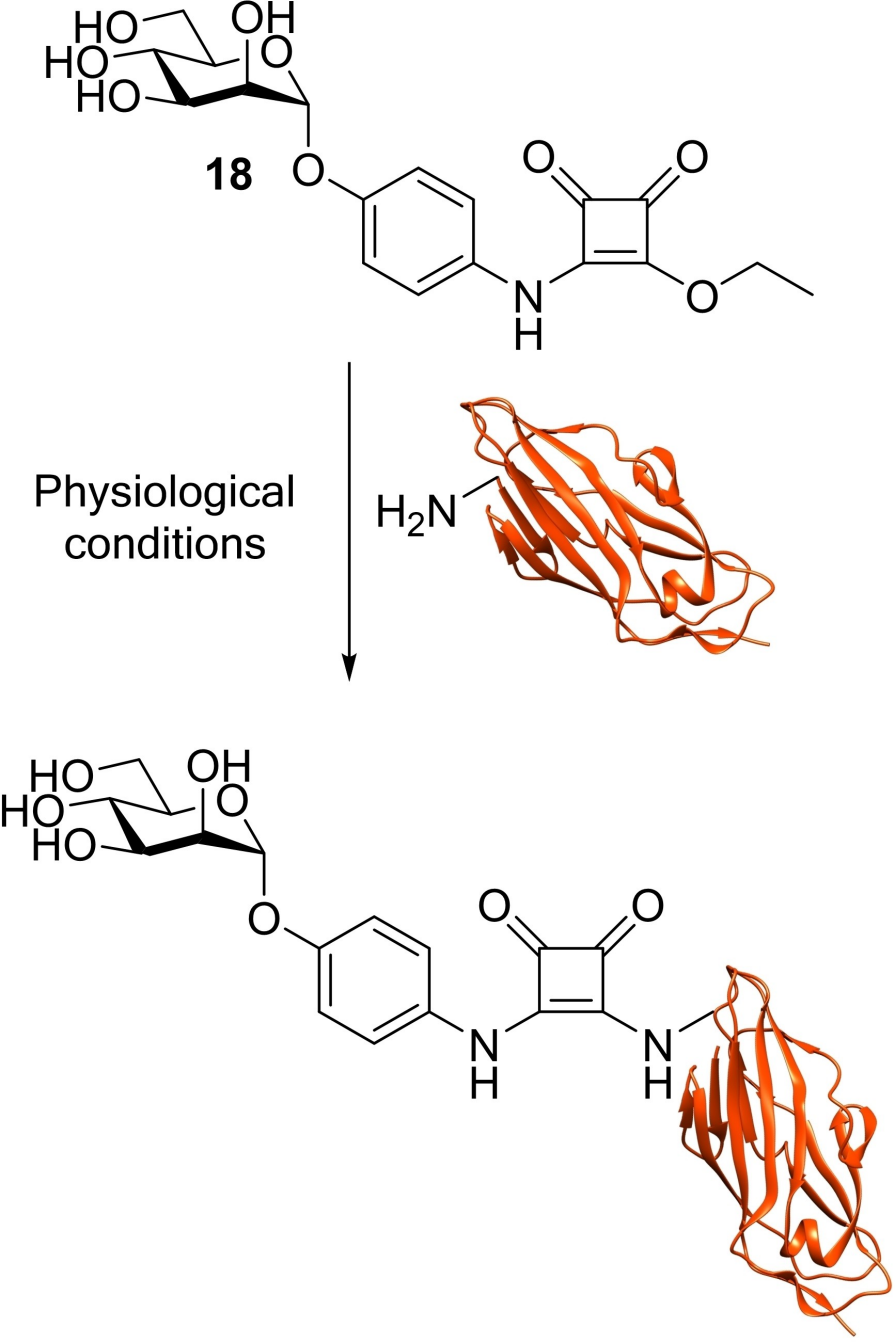
Reaction scheme for proposed covalent bond formation between squarate mannoside **18** and the N terminus of the FimH lectin (residue Phe1).

Following the discovery of squarate mannosides as potent FimH inhibitors^[64]^ it was suggested that the high potency observed with these ligands could be the result of covalent bond formation between the squarate mannoside **18** and the FimH binding site (Scheme 3). To see if this type of reaction was possible, squarate mannoside **18** was reacted with a l‐phenylalanine ester under physiological conditions. The reaction was shown to be successful.^[68]^

**Figure 19:**
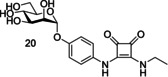
Structure of diamide squarate mannoside **20**.

However, diamide squarate mannoside **20** (Figure 19) was also synthesized and its structure prevents covalent crosslinking to FimH, yet was shown to be a potent inhibitor of FimH (IC_50_=6.38 μM±3.7 compared to nitrophenol IC_50_=274 μM±110). This suggests that the potency of squarate mannosides cannot be attributed to covalent crosslinking with the FimH binding site.^[68]^

**Figure 20:**
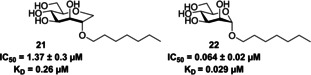
Structure, IC_50_ values and *K*_D_ values of 2‐*O*‐*n*‐heptyl‐1,6‐anhydro‐d‐glycero‐d‐galactitol **21** and *n*‐heptyl α‐d‐mannopyranoside **22**.^[69]^

Septanoses (Table 1, entry 5) were introduced by Ernst and co‐workers^[69]^ following the observation that methyl β‐septanosides bound to the jackbean lectin concanavalin A (ConA; another mannose‐selective lectin).^[69]^ Ernst and co‐workers^[69]^ used a competitive binding assay and ITC experiments to assess the potency of multiple septanose analogues on FimH_LD_. The results of these experiments showed 2‐*O*‐*n*‐heptyl‐1,6‐anhydro‐d‐glycero‐d‐galactitol **21** (Figure 20) displayed a ten‐fold lower potency for FimH than *n*‐heptyl α‐d‐mannopyranoside **22** (Figure 20; IC_50_=1.37±0.3 vs. 0.064±0.02 μM, *K*_D_=0.26 vs. 0.029 μM).^[69]^ Further investigations showed that while 2‐*O*‐*n*‐heptyl‐1,6‐anhydro‐d‐glycero‐d‐galactitol **21** (Figure 20) establishes the same hydrogen bonding network at the FimH binding site as *n*‐heptyl α‐d‐mannopyranoside **22** (Figure 20),^[69]^ the formation of this network results in a loss of conformational flexibility, causing a loss of entropy.^[69]^

**Figure 21:**
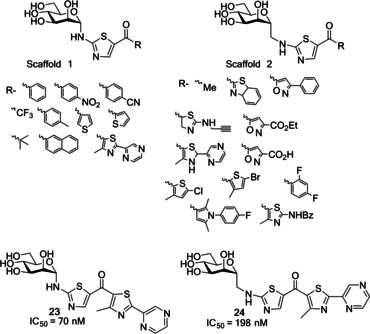
Structure of thiazolylaminomannosides scaffold **1** and neothiazolylaminomannoside scaffold **2** as well as the structure and IC_50_ values of thiazolylaminomannosides **23** and neothiazolylaminomannoside **24**.^[70a]^

Gouin and co‐workers developed a further class of d‐mannopyranoside‐based inhibitors: thiazolylaminomannosides (TazMan) and neothiazolylaminomannosides (NeoTazMan; Table 1 entry 6).^[70]^ A series of TazMan analogues were originally developed based on scaffold 1 (Figure 21); with the most potent analogue, thiazolylaminomannoside **23**, being 100 times more potent at preventing adherent‐invasive *E. coli* (AIEC) attaching to the intestinal cells compared to *n*‐heptyl α‐d‐mannopyranoside **22** (Figure 21).^[70b]^ Despite thiazolylaminomannoside **23** displaying high *in vitro* potency, it showed limited *in vivo* efficiency, likely due to having low pH stability and low water solubility; this restricts the applications of thiazolylaminomannoside **23**.^[70b]^ To combat these poor *in vivo* results, Gouin and co‐workers synthesized a second neothiazolylaminomannoside (NeoTazMan) series based on scaffold **2** (Figure 21).^[70a]^ Thiazolylaminomannoside **23** NeoTazMans counterpart neothiazolylaminomannoside **24** (Figure 21) was shown to have improved *in vivo* properties, being stable to both enzymatic and acid hydrolysis. However, neothiazolylaminomannoside **24** was shown to be 2.8 times less potent than thiazolylaminomannoside **23** with an IC_50_ of 194 versus 70 nM.^[70a]^ These initial investigations show that both TazMan and NeoTazMan are effective FimH inhibitors. However, it is unknown how applicable these analogues will be as therapeutics in the treatment of UPEC‐induced UTIs, as existing studies have only explored their applications in the treatment of Crohn's disease.^[70]^

**Figure 22:**
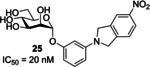
Structure and IC_50_ value of indolinylphenyl **25**.^[71]^

The final class of α‐d‐mannopyranoside‐based inhibitors discussed in this review are indolynylmannosides (Table 1 entry 7), which were first investigated by Ernst and co‐workers.^[71]^ Ernst and co‐workers synthesized a series of indolylphenyl and indolinylphenyl α‐d‐mannosides and then investigated their inhibitory and pharmacokinetic properties. The most promising analogue was indolinylphenyl **25** (Figure 22) achieving an IC_50_ of 20 nM.^[71]^ Administering a low dose of indolinylphenyl **25** (1 mg/kg) in a mouse model achieved a minimum inhibitory concentration in the bladder for >8 h.^[71]^ Furthermore, a 1 mg/kg dose of indolinylphenyl **25** was shown to reduce colony‐forming units in the bladder by a factor of 3.7 compared to untreated mice; this result is in line with the reduction seen in mice treated with ciprofloxacin (8 mg/kg).^[71]^

**Figure 23:**
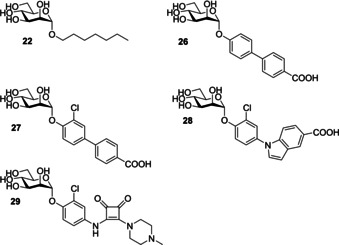
Structures of the α‐d‐mannopyranoside‐based inhibitors, *n*‐heptyl α‐d‐mannopyranoside **22**, biphenyl α‐d‐mannopyranoside derivatives **26** and **27**, indolylphenyl mannoside derivative **28** and squarate mannosides derivative **29**.

A factor that must be considered when designing mannose‐based inhibitors is selectivity, as humans possess other mannose binding lectins (e. g., human mannose binding proteins) meaning a lack of selectivity can result in off‐target reactions. Ernst and co‐workers^[72]^ have previously investigated the selectivity of five potent FimH antagonists (all with nM IC_50_ values (Figure 23) against eight human mannose receptors. If an analogue displayed 10^5^ times greater potency for FimH over the mannose binding proteins it was classified as selective, and assumed to not cause adverse effects due to non‐selective binding.^[72]^ All inhibitors tested were shown to have a 10^5^‐fold lower affinity for the human mannose binding proteins than FimH, confirming their selectivity.^[72]^ The FimH inhibitors have been optimized to contain hydrophobic substituents at their reducing end; these substituents can interact with the tyrosine gate located at the entrance of the FimH binding site. The tyrosine gate is a feature unique to FimH, which likely explains the selectivity displayed by these inhibitors.^[72]^ Furthermore, multivalent ligand presentation is known to be hugely important in nature, and as the inhibitors investigated here are only capable of monovalent binding they likely only display low affinity for human mannose receptors.^[72]^

### 2.3 Use of polyvalent mannose scaffolds in FimH

**Figure 24:**
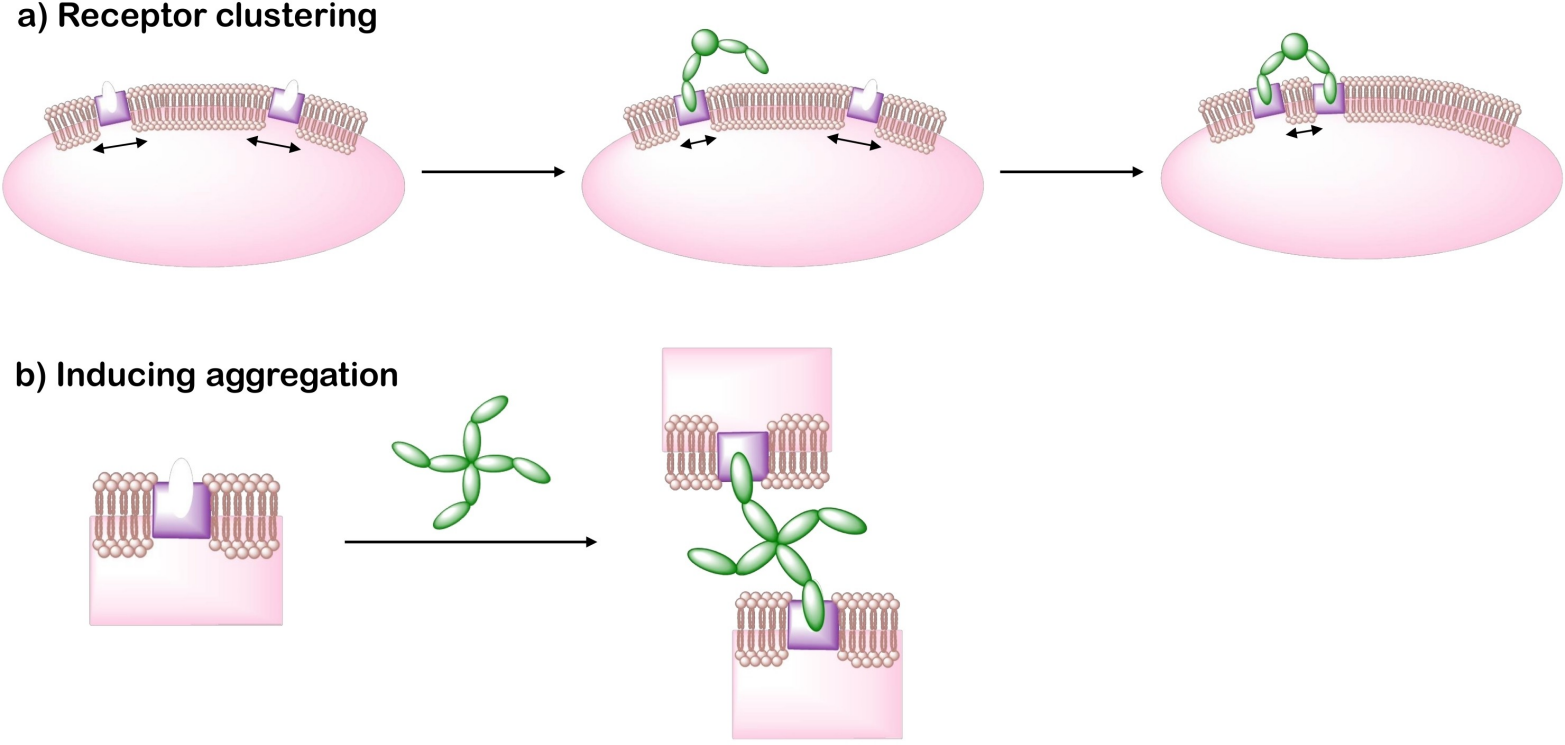
Diagram showing two potential mechanisms whereby multivalent ligands can increase apparent binding affinity. a) Clustering effect where a multivalent ligand binds to one receptor initially and then captures additional receptors as they diffuse into close proximity resulting in clustering of the ligand ‐bound receptors. b) Induced aggregation whereby multivalent ligands bind to multiple lectins on different bacterial units cross linking them together.

In nature carbohydrate ligand presentation is mainly multivalent,^[72]^ thus suggesting that the potency of α‐d‐mannopyranoside‐based inhibitors may be limited due to only achieving monovalent targeting. The phenomenon describing how many multivalent ligands display greater affinity than their monovalent counterparts has been termed the “cluster glycoside effect”.^[73]^ Multivalent ligand presentation can increase lectin binding via multiple factors, including receptor clustering and crosslinking.^[74]^ Receptor clustering occurs when monovalent lectins/ligands are anchored to the cell membrane. The presence of a multivalent binding species can capture receptors freely diffusing in the membrane, thus inducing receptor clustering (Figure 24a). However, this mechanism is unlikely to occur in the bacterial outer membrane (OM) due to the restricted lateral diffusion of OM proteins.^[75]^ Receptor clustering may occur by default in the bacterial OM near sites of beta‐barrel transmembrane protein insertion (i. e. BAM complex will mediate insertion of FimD) due to the restricted lateral diffusion. As a consequence, the transmembrane FimD usher protein may already be present at the extracellular surface in clusters.^[74]^ Multivalent ligand presentation can further increase lectin binding through crosslinking. Here, individual ligands on a multivalent species bind to lectins on separate target units, crosslinking them and aggregating bacteria together (Figure 24b).^[74]^ Due to the complexity of multivalent carbohydrate interactions, it is often an accumulation of multiple factors that cause the “cluster glycoside effect”. In the case of FimH it is likely that multivalent mannose‐based ligands bind to multiple different FimH units, resulting in the formation of *E. coli* clusters. Multivalent glycomaterials attached to filters have been used to aggregate *E. coli* and remove them from solution.^[76]^

There has been substantial research into the development of FimH‐targeting polyvalent mannose scaffolds, with applications in the treatment of UPEC‐induced UTIs. Multiple different polyvalent mannose scaffolds have been investigated with three of the major ones being multimeric heptylmannosides,^[77]^ glycoclusters^[78]^ and dendrimers.^[79]^

**Figure 25:**
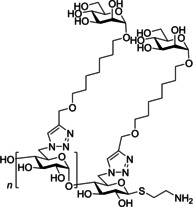
Structure of multivalent HM glycoconjugate series designed using a carbohydrate core where n=0, 1,2 and 6 (when using β‐cyclodextrin core).

One of the most researched polyvalent scaffolds are multimeric heptylmannosides (Table 2); these aim to build upon the high‐inhibitory potency displayed by heptyl α‐d‐mannoside (HM; Figure 5). Gouin and co‐workers synthesized an initial series of multivalent glycoconjugates (Table 2, entry 1) based on a 1,1,1‐tris(hydroxymethyl)ethane or pentaerythritol core and measured their inhibitory potencies using a HAI‐assay and a bladder binding assay (BBA) with human bladder cell line 5637.^[77a]^ Both the HAI titre and the BBA data showed a positive correlation between potency and valency.^[77a]^ A second multivalent HM glycoconjugates series (Figure 25, Table 2, entry 2) was designed based on a carbohydrate core, with one heptamannoside analogue tethered to a ring‐opened β‐cyclodextrin.^[77b]^ Binding affinity of this series was examined using HAI titre and ITC measurements. As with the previous series, HAI titre measurements showed a positive correlation between valency and potency, with the heptavalent opened β‐cyclodextrin glycoconjugate achieving a titre in the nanomolar region (60 nM).^[77b]^ Though initial ITC measurements supported this trend, latter measurements varied, likely due to increased calorimetry noise reducing the accuracy of the *K*_d_ measurements.^[77b]^ Two further heptavalent β‐cyclodextrin‐linked HM glycoconjugates (Table 2, entry 3) were synthesized using two different spacer lengths. Their biophysical properties were assessed using ITC, extracting *K*_d_ values and molar ratios. Both heptavalent β‐cyclodextrin‐linked glycoconjugates were significantly more potent than their monovalent counterparts, with *K*_d_ values in the nanomolar range.^[77c]^ Reverse titration measurements showed that the use of a shorter spacer unit led to superior potency (*K*_d_=2.9 nM±0.03) compared to the use of a longer linker (*K*_d_=33.0 nM±6.6), despite only achieving partial binding occupancy (molar ratios=3.01±0.03 for the short spacer and=7.7±0.06 for the longer spacer).^[77c]^ This suggests potency is not solely dependent on valency. The *in vivo* properties of both heptavalent β‐cyclodextrin‐linked glycoconjugates were assessed using a murine cystitis model with C3H/HeN mice. Both heptavalent glycoconjugates were shown to be 100 times more potent than their monovalent counterparts.^[77c]^

**Table 2:** Summary of the multivalent heptamannoside structure discussed.

	Core unit	Ref.
1)	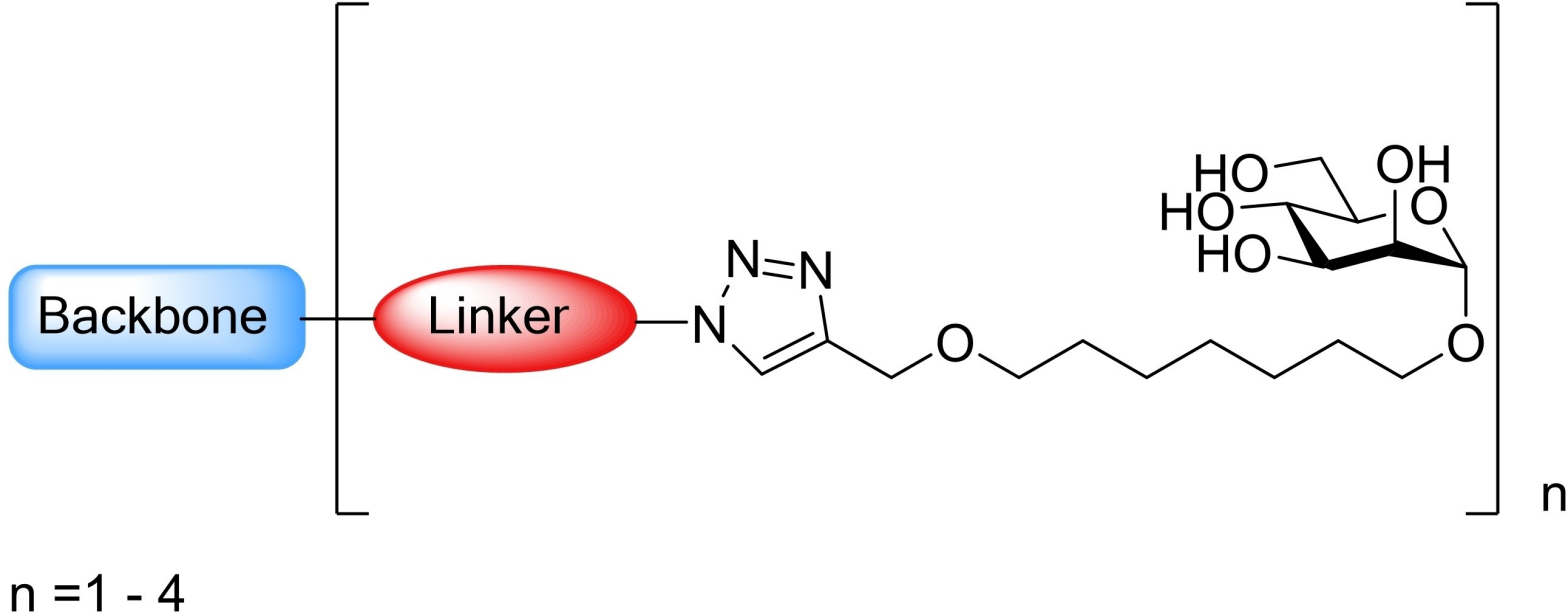	^[77a]^
2)	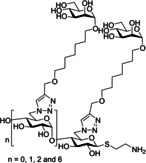	^[77b]^
3)	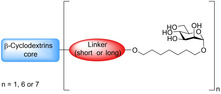	^[77c]^
4)	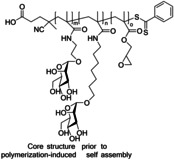	^[77e]^
5)	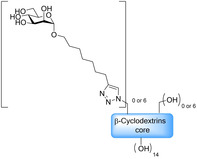	^[77d]^

Further work into development of a multivalent heptylmannoside has investigated the design and use of glyconanoparticles^[77e]^ (Table 2 entry 4) and multimeric heptyl‐mannosides^[77d]^ (Table 2 entry 5) in targeting adherent invasive *E. coli* (AIEC), a bacterial strain present in the ileal lesion of Crohn's disease patients.^[77d]^

Mannose‐based glycoclusters are a further polyvalent mannose scaffold that has been investigated. Multiple different glycocluster series have been synthesized using a variety of different backbone scaffolds (Table 3); for example, cysteine residues (Table 3, entry 1),^[78a]^ functionalized pentaerythritol (Table 3, entry 2),^[78b]^ thiourea‐bridged clusters (Table 3, entry 3),[^78c^, ^80^] peptide‐bridged clusters (Table 3, entry 4)^[80]^ and carbohydrate centred clusters (Table 3, entry 5).^[78d]^ The *in vitro* efficacy of different glycocluster series have been assessed using a variety of methods; e. g. IC_50_ measurements, inhibition of baker's yeast agglutination assay, HAI titre, and ELISA. Generally, a positive correlation between potency and valency was observed. However, other factors such as scaffolds structure (e. g., inclusion of a phenyl unit in the scaffold) were shown to significantly contribute to potency.

**Table 3:** Summary of the organic scaffold used in the synthesis of mannose‐based glycoclusters.

	Organic scaffold	Structure	Ref.
1)	cysteine	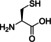	^[78a]^
2)	azide‐ or alkyne‐bearing pentaerythritols	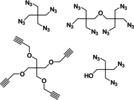	^[78b]^
3)	thiourea‐bridged clusters	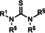	[^78c^, ^80^]
4)	peptide‐bridged clusters	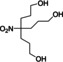	^[80]^
5)	carbohydrate centred cluster	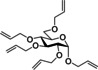	^[78d]^

Investigations have also focused on the synthesis of a mannose‐bearing dendrimer.^[79]^ Roy and co‐workers synthesized multiple series of mannose‐bearing dendrimers using different scaffolds. One scaffold of particular note was the α‐amino‐l‐lysine scaffold, from which they synthesized a dendrimer containing 16 mannose units with a potency 500 times greater than methyl mannose (HAI titre=1.0 vs. 500 μM).^[79b]^

Diamond nanoparticles can also serve as a polyvalent mannose scaffold. Mannose‐functionalized diamond nanoparticles where originally applied to the detection and removal of *E. coli* from bacteria‐polluted water.^[76b]^ More recently mannose‐functionalized diamond nanoparticles have been demonstrated to be potent *E. coli* anti‐adhesives, displaying impressive potency in a bladder cell adhesion assay (RIP=9259 vs. 1 for methyl mannose)^[81]^ as well as reducing *E. coli* biofilm formation.^[81]^

A final scaffold discussed here is fullerenes. Copper‐catalysed click chemistry was used to functionalize fullerenes with mannose moieties,^[82]^ yielding three fullerene analogues bearing 12 mannoside units.^[83]^ ITC and SPR showed these polyvalent analogues to be capable of multivalent binding and found that both polyvalent and monovalent analogues displayed a *K*_d_ in the nanomolar range.^[83]^ A further HAI‐ assay was performed which found the polymeric fullerenes to be between 2.8–30 times more potent than their monomeric counterparts.

Other polyvalent mannose scaffolds reported include glycopeptides,[^79d^, ^84^] polyvalent nanoparticles^[85]^ and pillar[5]arene derivative.^[86]^ Research into these scaffolds is in its infancy but initial *in vitro* investigations appear promising.

Oral administration is the preferred route for drug administration, and as such the applications of any analogue which cannot be orally administered are limited. Due to their large structure and high hydrogen bond donor and acceptor content, polyvalent mannosides are unlikely to be orally absorbed. This could severely limit their applications in the treatment of UTIs but also makes them potentially powerful candidates in the treatment of AIEC‐induced Crohn's disease.[^77d^, ^77e^] Indeed clinical trials are currently being performed on the use of multivalent inhibitor Sibofimloc (TAK‐018/EB8018) in the treatment of Crohn's disease.[^87^, ^88^]

In this review we have focused on discussing the advances being made towards the development of mannose‐based UPEC targeting UTI treatments. It is worth noting that research has also been done to develop galactose analogues capable of inhibiting the F9 pilus, a homologue to the type one pilus, which has an important role in the maintenance of UPEC‐induced UTIs.^[89]^ While it is not the aim of this review to cover advances in the development of these galactose‐based inhibitors, interested readers can find information on this topic published elsewhere[^89^, ^90^].

## 3 Summary and Outlook

We have summarized the main areas of interest regarding research into mannose‐based FimH inhibitors. In the current climate, where antibiotic resistance is becoming ever more prevalent, there is a need to develop effective targeted antibacterial treatments. Overall the studies discussed illustrate the great potential of mannose‐based inhibitors as targeted treatments against UPEC‐induced UTIs. Analogues from each class of mannose‐based inhibitor have shown impressive potency against FimH, and warrant further investigation. However, there are disadvantages associated with the use of each inhibitor class. Mannose‐capped oligosaccharides most accurately reflect the structure of natural FimH ligands, yet their likely lack of oral bioavailability and complex chemical synthesis widely restricts their use. Simpler α‐d‐mannopyranoside‐based inhibitors are currently the most explored class of mannose‐based FimH inhibitors and potentially the most promising. The main advantages of α‐d‐mannopyranoside inhibitors are their simpler and smaller structures, making them easier to synthesize and providing a better chance of oral absorption. However, the development of analogues with both high *in vivo* efficacy and high oral bioavailability is challenging. A further limitation to α‐d‐mannopyranoside‐based inhibitors is that their structure prevents polyvalent FimH targeting, potentially limiting their potency. Research into polyvalent mannose scaffolds is still largely in its infancy, and it is not known as yet how effective these inhibitors will be. The potential applications of a high‐affinity polyvalent α‐d‐mannopyranoside‐based inhibitor are vast, yet their high molecular weight and hydrophilic properties could impact their oral bioavailability, preventing them from functioning as an effective UPEC‐induced UTI treatment. These analogues may find alternative applications as a potential treatment for Crohn's disease. It is expected that continued work into both established and novel FimH targeting methods will further advance the field, and take us closer to the development of an effective treatment for UPEC‐induced UTIs.

## Conflict of interest

The authors declare no conflict of interest.

## Biographical Information

*Natasha Hatton received her MChem degree in chemistry from the University of Liverpool in 2017. She is currently engaged in Ph.D. research at the University of York, under the co‐supervision of Drs. Martin Fascione, Christoph Baumann and Laurence Wilson. Her work focuses on the synthesis and applications of mannose‐based glycoconjugates*.

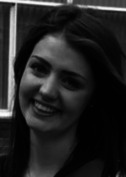

## Biographical Information

*Christoph Baumann received his Ph.D. from the University of Minnesota in Minneapolis* – *St. Paul (USA). Under the guidance of Professor Victor Bloomfield, he used single‐molecule experiments to show that multivalent cations can influence DNA elasticity. During a postdoctoral period at the University of York (UK, 1998*–*2003) with Drs. James Hoggett and Justin Molloy, he developed a novel single‐molecule biophysical assay for DNA transcription by RNA polymerase. He took up a lectureship at York in 2003, and more recently has been using single‐molecule fluorescence approaches to visualise the spatio‐temporal dynamics of proteins and lipids in bacterial cell membranes*.

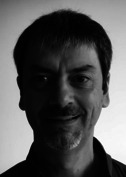

## Biographical Information

*Martin Fascione received his Ph.D. from the University of Leeds in 2009, working under the supervision of W. Bruce Turnbull on the stereoselective synthesis of 1,2‐cis‐glycosides. Following a postdoctoral period in Leeds, he was awarded a Marie Curie International Outgoing Fellowship to study the mechanisms of carbohydrate‐processing enzymes with Profs. Steve Withers, FRS, at UBC (Vancouver, Canada, 2012*–*2013) and Gideon Davies, FRS, FMedSci, at the University of York (2013*–*2014). In 2014 he took up a lectureship in the York Structural Biology Laboratory, within the Department of Chemistry, focussing upon deciphering the roles that carbohydrates play in the etiology of disease and applying this knowledge in the development of innovative new therapeutics*.

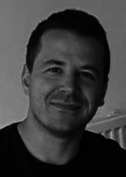

## Supporting information

As a service to our authors and readers, this journal provides supporting information supplied by the authors. Such materials are peer reviewed and may be re‐organized for online delivery, but are not copy‐edited or typeset. Technical support issues arising from supporting information (other than missing files) should be addressed to the authors.

Supplementary
