# Supplementary material for: Developments in Mannose‐Based Treatments for Uropathogenic Escherichia coli‐Induced Urinary Tract Infections
Source: Chembiochem. 2020 Nov 2;22(4):613–29. doi: 10.1002/cbic.202000406 (PMC7894189; doi:10.1002/cbic.202000406)
Supplement: Supplementary file 1 — Supplementary [file CBIC-22-613-s001.pdf]

Author Contributions

N.H. Writing – original draft:Lead; Writing – review & editing:Lead
C.B. Supervision:Lead; Writing – review & editing:Lead
M.F. Writing – original draft:Supporting; Writing – review & editing:Lead
